# Guiding cell migration in 3D with high-resolution photografting

**DOI:** 10.1038/s41598-022-11612-y

**Published:** 2022-05-23

**Authors:** Simon Sayer, Tommaso Zandrini, Marica Markovic, Jasper Van Hoorick, Sandra Van Vlierberghe, Stefan Baudis, Wolfgang Holnthoner, Aleksandr Ovsianikov

**Affiliations:** 1 Research Group 3D Printing and Biofabrication, Institute of Materials Science and Technology, TU Wien, Vienna, Austria; 2 Austrian Cluster for Tissue Regeneration (https://www.tissue-regeneration.at), Vienna, Austria; 3 Polymer Chemistry and Biomaterials Group, Centre of Macromolecular Chemistry, Department of Organic and Macromolecular Chemistry, Ghent University, Ghent, Belgium; 4 Polymer Chemistry and Technology Group, Institute of Applied Synthetic Chemistry, TU Wien, Vienna, Austria; 5 Ludwig-Boltzmann-Institute for Traumatology, The Research Centre in Cooperation with AUVA, Vienna, Austria

**Keywords:** Cell biology, Chemical biology, Stem cells, Chemistry, Materials science

## Abstract

Multi-photon lithography (MPL) has proven to be a suitable tool to precisely control the microenvironment of cells in terms of the biochemical and biophysical properties of the hydrogel matrix. In this work, we present a novel method, based on multi-photon photografting of 4,4′-diazido-2,2′-stilbenedisulfonic acid (DSSA), and its capabilities to induce cell alignment, directional cell migration and endothelial sprouting in a gelatin-based hydrogel matrix. DSSA-photografting allows for the fabrication of complex patterns at a high-resolution and is a biocompatible, universally applicable and straightforward process that is comparably fast. We have demonstrated the preferential orientation of human adipose-derived stem cells (hASCs) in response to a photografted pattern. Co-culture spheroids of hASCs and human umbilical vein endothelial cells (HUVECs) have been utilized to study the directional migration of hASCs into the modified regions. Subsequently, we have highlighted the dependence of endothelial sprouting on the presence of hASCs and demonstrated the potential of photografting to control the direction of the sprouts. MPL-induced DSSA-photografting has been established as a promising method to selectively alter the microenvironment of cells.

## Introduction

Hydrogels are constituted of crosslinked hydrophilic polymer chains, with a high swelling ratio and permeability. The mechanical characteristics of hydrogels are similar to soft tissue and they are permeable to oxygen, nutrients and other soluble metabolites, making them a suitable material to create scaffold-based 3D cell culture systems^1^. They are providing an environment that facilitates cell–cell and cell–matrix interactions by assuming the role of the extracellular matrix (ECM). This reduces the difference between the physiological environment and traditional cell cultures^2^. Besides providing a homogeneous platform in which cells are behaving more similarly to the natural conditions, subsequently improving the transferability of in vitro research, in areas such as drug development, to the actual in vivo effect, advances in techniques relying on the local modification of the cell-laden matrix allow for the adaption of the individual cell’s microenvironment^3,4^. Hydrogels can be derived from a variety of different natural or synthetic polymers^5^. Among these materials, gelatin methacryloyl (gel-MA) is a promising naturally derived hydrogel precursor that is photopolymerizable, biocompatible, susceptible to enzymatic cleavage, supports cell adhesion, has tunable mechanical properties and is abundantly available^6–8^.

The ECM is a key aspect of the cell’s microenvironment and can differ in terms of the composition and the biochemical (e.g. chemical functional groups) and biophysical parameters (e.g. mechanical stiffness, degradability)^9^. In vitro studies have shown that these factors influence cell behavior and functions, such as migration^10^, proliferation^11,12^ differentiation^13^ and gene and protein expression^14^.

During embryonic development, wound healing and tumor growth, new blood vessels are formed from existing ones. This process, called angiogenesis, relies on cell–matrix interactions and the remodeling of the ECM^15^. The physico-chemical properties of the matrix as well as the entire microenvironment play a crucial role in the regulation of angiogenesis^16^. A functioning blood vessel network is necessary to maintain homeostasis and facilitate the exchange of oxygen, nutrients, biochemicals and waste. Moreover, it seems that it also acts as a structural template and thereby influences the organization of tissue^17,18^. The diffusion range of oxygen (100–200 µm) is the limiting factor to the size of implantable engineered tissue^19,20^. Although the host vasculature will generally grow into the tissue and form a network, this process can be very slow, subjecting parts of the tissue to oxygen and nutrient deficiency for a size-dependent period^21,22^. Despite the importance of vascularization in tissue engineering, it remains one of the greatest challenges in the field^23^.

Besides the natural organization of endothelial cells, which relies on remodeling after implantation and most likely offers no clear area for anastomosis, there are many different approaches to control vascularization^24^. These approaches range from different patterning techniques (i.e. the formation and subsequent seeding of hollow channels, photo-micropatterning, 3D bioprinting or electrospinning), to cell guiding approaches, where either biochemical signals, mechanobiological cues or a spatial inhomogeneity of the scaffold’s material properties, are responsible for the preferential migration and sprouting of endothelial cell co-cultures^12,25–30^. Further areas of application for the fabrication of in vitro vessels, besides implantable tissue, are organs-on-a-chip^31^, platforms for studying blood vessel formation under physiological^32^ and pathological conditions^33^ and setups for observing endothelial cell functions and interactions^34^.

The human vasculature is a complex, hierarchical system with a feature size that ranges over three orders of magnitude. Arterioles, capillaries and venules are the constituents of what is called microcirculation. Capillaries, the smallest and most numerous blood vessels in the body, have an inner diameter between 5–10 µm and a very thin vessel wall^35^. Some vascular tissue engineering techniques, such as sheet rolling and tubular molding, are not suitable for the fabrication of vessels at the necessary micrometer scale^36^. Others, such as embedded bioprinting^37^, inkjet bioprinting^38^ or extrusion bioprinting^39^ are capable of producing vessels at a spatial resolution that is sufficient for arterioles and venules but not for capillaries and are limited in terms of 3D control. The biofabrication of microvessels is therefore reliant on biocompatible techniques that allow for the creation of complex structures at a sufficiently high resolution.

In general, light-based patterning techniques can provide a solution with their ability to create structures at relevant sizes for capillaries, venules and arterioles (5–100 µm) and their comparatively high cell viability^36^. Laser-induced forward transfer (LIFT)^40^ and digital light processing (DLP)^41^ meet those criteria, but they are limited in their ability to create fully arbitrary 3D structures and cannot match the high resolution of multi-photon lithography (MPL). Various soft lithography approaches allow for the creation of patterns at the micro- and nanometer scale, such as nanoimprint lithography^42^ or replica molding^43^. It is possible to use these methods for the fabrication of microvessels, however, due to the limitation to topographical features, the necessary freedom in design and spatial positioning is lacking^44^.

Multi-photon lithography, a process in which a femtosecond (fs) laser beam is focused through an objective, can initiate a photochemical reaction at subdiffractional resolution, in an arbitrary 3D manner within materials that are transparent at the applied laser wavelength. The high resolution and 3D-nature of the process are achieved by the non-linear relation between the laser intensity and the probability of multi-photon absorption. Dobos et al. (2020) recently reported high-definition bioprinting of microvascular structures directly within a microfluidic chip using MPL^30^. In this approach, a relatively large volume of a hydrogel precursor, compared to the resulting volume of branching channels, has to be cross-linked. Therefore, a process aimed directly at patterning pre-formed hydrogels would be much more efficient throughput-wise. Various MPL approaches are applicable to already formed structures. Hydrogels can be patterned with chemical functionalities, by covalent cross-linking^44^ or photocaging^45^, previously formed cross-links can be degraded^46^ and the material can be cross-linked or densified upon multi-photon absorption^47^. While these approaches are suitable in terms of resolution, complexity and biocompatibility, they all require materials with special functional groups. Processes based on photoablation, which is the degradation of a material through the photolysis of non-specific chemical bonds, extreme local heating or microcavitation, are the only exceptions. However, they require light with high energy and intensity, which has potentially adverse effects on cells and the integrity of the surrounding material^48,49^.

To overcome all these difficulties and side effects, we present a straightforward, biocompatible, high-resolution approach to alter the chemical and physical properties of preformed hydrogels in 3D. The presented approach has various advantages over other micropatterning techniques. It is straight-forward, universally applicable to any transparent hydrogel, without the need for materials with special functional groups, works with commercially available materials and allows for the patterning in the presence of cells. Due to the preceding cell embedding step, the sedimentation of cells during the patterning process is prevented. A homogeneous cell distribution is therefore assured throughout the entire process. This method is based on molecular grafting, which is often used to immobilize molecules on a substrate’s surface^50,51^. Utilizing multi-photon grafting, first proposed by Ovsianikov et al. (2012), allows for the 3D localized introduction of a molecule into the polymer backbone of a hydrogel, eliminating the surface constraint^52^. The selection of a commercially available, azido groups-containing and more importantly cytocompatible grafting agent allowed us to translate this process to cell-laden hydrogels, and to observe its influence on cell behavior. Furthermore, a high laser scanning speed of 1000 mm/s ensures a fast patterning process at a high resolution in 3D.

While MPL has been used to induce cellular alignment through local post-polymerization^47^, it is novel to guide cells by the local introduction of a molecule into the polypeptide backbone of a cell-laden hydrogel matrix. We report for the first time the application of multi-photon photografting, in which the spatiotemporal modifications of a cell-laden hydrogel matrix lead to the alignment and directional migration of cells. Patterns with a feature size of 10 × 10 µm^2^ were photografted in a gelatin-based matrix and the preferential alignment of human adipose-derived stem cells (hASCs) was observed over the course of 13 days. Photografted 3D patterns around spheroids were shown to induce directional migration of hASCs into the modified regions of the hydrogel. By utilizing co-culture spheroids of hASCs and human umbilical vein endothelial cells (HUVECs), we were able to demonstrate the potential of MPL to guide the collective migration of endothelial cells in the form of a sprout.

## Materials and methods

### Synthesis of lithium (2,4,6-trimethylbenzoyl) phenylphosphinate (Li-TPO-L)

The photoinitiator lithium (2,4,6-trimethylbenzoyl)phenylphosphinate (Li-TPO-L) was prepared according to a published protocol (Markovic et al. 2015)^53^. A working concentration of 0.6 mM has been chosen in accordance with the cell encapsulation study by Markovic et al. (2015)^53^.

### Cell culture

hTERT immortalized human adipose-derived mesenchymal stem cells (hASCs/TERT1, Evercyte) were either used as purchased or retrovirally infected with green-fluorescent proteins (GFP-hASCs) according to the protocol by Knezevic et al. (2017)^54^. Endothelial Cell Growth Medium-2 (EGM-2) was prepared by supplementing the EBM-2 Basal Medium with the EGM-2 SingleQuots Supplements from the EGM-2 Endothelial Cell Growth Medium-2 BulletKit (Lonza). hASCS/ GFP-hASCs were cultured in T-75 cell culture flasks (Greiner Bio-One) in EGM-2 supplemented with 10% fetal calf serum (FCS, Sigma-Aldrich). Human umbilical vein endothelial cells (HUVECs, PELOBiotech GmbH) were infected with red-fluorescent protein (RFP)-expressing lentiviral particles at passage 1. Zeocing resistant RFP-HUVECs were selected and cultured in EGM-2 supplemented with 5% FCS in T-25 cell culture flasks (Greiner Bio-One). All cell lines were cultivated in an incubator (BINDER) at 37 °C with 5% CO2 in a humid atmosphere and split at around 80 percent confluency. The media were changed every 2 to 3 days.

### Preparation of methyl cellulose stock solution

For the preparation of methyl cellulose, 600 mg of methylcellulose powder (Sigma-Aldrich) was weighed, transferred to a magnetic stirrer and autoclaved (VWR Vapour-LineECO). 50 mL of EGM-2 medium was heated to 60 °C and added to the methylcellulose powder. The solution was then stirred for 20–30 min at room temperature and afterward for 2 h at 4 °C. The stirred methylcellulose solution was transferred to a falcon tube and centrifuged for 2 h at 5000 g and 4 °C. The clear, highly viscous supernatant was transferred to a new falcon tube and stored as the methyl cellulose stock solution at 4 °C.

### Preparation of agarose molds and spheroids

The spheroids were formed using MicroTissues 3D Petri Dish micro-molds (Sigma-Aldrich) casted with a 2% agarose solution. This solution was created by autoclaving 1 g agarose powder (Sigma-Aldrich) and dissolving it in 50 mL sterile water. The heated agarose solution was pipetted into the micro-mold and left for a couple of minutes to ensure physical gelation. The molds were transferred to a 12-well plate and soaked in 3 mL PBS for 30 min for equilibration. Each agarose mold has 81 microwells.

HUVECs and hASCs were detached from the cell-culture flask by washing them twice with PBS and then exposing them to Trypsin–EDTA 0.25% (Gibco) for 5 min. Medium was added after successful detachment for neutralization. The cells were counted and a solution containing 40,500 HUVECs and 40,500 hASCs was aliquoted. After spinning the cells down in a benchtop centrifuge (Rotina 380 R, Hettich), the remaining solution was carefully removed and the cells were resuspended in 190 µl of medium containing 20% methyl cellulose. After carefully mixing the media, to ensure an even distribution of cells, it was pipetted into the agarose mold. The 12-well plate was incubated for 1 h, so that the cells could gather in the microwells, before carefully adding 2 mL of 80% EGM-2 5% and 20% methyl cellulose stock solution. After 24 h of incubation, the spheroids were checked under a brightfield microscope, the methylcellulose medium was removed, fresh medium was added and the spheroids were harvested.

### Methacrylation of µ-dish with glass bottom

In a first step, the methacrylation solution was prepared by mixing 48 mL ethanol, 0.3 mL acetic acid and 50 mL deionized water with a magnetic stirrer. After having slowly added 2 mL of 3-(trimethoxysilyl)propyl methacrylate dropwise, the solution was stirred for 30 min and transferred to the plasma-cleaned µ-dishes with a glass bottom (ibidi), covering the glass bottoms for 15 min. The methacrylation solution was disposed and the µ-dishes were washed with deionized water. To evaporate the remaining liquid, the µ-dishes were placed in an oven for 30 min at 110 °C.

### Preparation of hydrogel pellets from gel-MA

Gel-MA with a degree of substitution (DS) of 63% was synthesized according to a published protocol^55,56^. The DS was confirmed using proton nuclear magnetic resonance (^1^H NMR) spectroscopy. A 10 wt% stock solution was prepared by dissolving gel-MA in phosphate-buffered saline (PBS, Sigma-Aldrich). This stock solution was mixed with the photoinitiator Li-TPO-L (working solution of 0.6 mM) and co-culture spheroids suspended in EGM-2 supplemented with 5% FCS at an appropriate ratio to achieve a gel-MA concentration of 5 wt%. The solution was mixed thoroughly to ensure an even distribution of spheroids. One mold containing 81 spheroids was used for 100 µL of hydrogel precursor solution. The actual number of spheroids encapsulated in the hydrogel pellet depends on the success rate of the transfer from mold to pellet. 30 µL of the solution was pipetted onto a methacrylated glass bottom dish and placed in the UV curing chamber (Emission dose of 1 J over 4.5 min, 365 nm, UV Crosslinker AH, Boekel Scientific). After crosslinking, 2 mL of 1 mM 4,4′-diazido-2,2′-stilbenedisulfonic acid (DSSA, Sigma-Aldrich), dissolved in EGM-2 supplemented with 5% FCS,was added. The pellets were incubated for 24 h and thereafter, transferred to the MPL setup to modify the chemical properties. Right after, the pellets were washed twice with medium to remove any residual DSSA.

### MPL setup and processing parameters

The MPL experiments are performed using a setup reported previously^57^. To communicate with the MPL setup, the software Think3D from UpNano GmbH was used^58^.

CAD models were sliced along the vertical direction, and the slices were scanned with parallel lines. The horizontal and the vertical line spacings were 0.5 µm and 1 µm, respectively. The orientation of the lines in either x- or y-direction was changed layer by layer, with an offset between the planes. Scanning speed was set to 1000 mm/s and the power ranged from 125 to 300 mW. An Olympus 10x/ 0.4 NA objective was used. The patterning direction was from top to bottom in order to prevent the laser from passing through already photografted regions. The setup is using a femtosecond laser with wavelength-tunable between 690 and 1040 nm. Based on preliminary results, wavelengths of 700 nm and 725 nm have been selected.

### Confocal laser scanning microscopy and image analysis

A confocal laser scanning microscope (LSM800, Carl-Zeiss) was used to obtain the images. DSSA was visualized by exciting at a wavelength of 410 nm, the hASCs were visualized by exciting at a wavelength of 488 nm and the HUVECs were visualized by exciting at a wavelength of 561 nm. The time-lapse imaging was performed under 37 °C and 5% CO2, in a humid environment. The migration distances were quantified using the ZEN Blue 2.6 software. More details on the quantification process are provided in the supporting information, including an image showcasing the measurement of the migration distance in Figure S4.

The fluorescent images of cell alignment were quantified using Fiji, which is an open-source image processing package based on ImageJ. Quantification was based on the fluorescent area to account for the potential overlapping of multiple cells. After thresholding the image, a watershed segmentation was performed. Ellipses were fitted to the particles and the angles between the primary axis and a horizontal line were analyzed. The area of particles within a deviation of 10° from the horizontal and vertical lines was counted as aligned.

### Statistical analysis

The statistical analysis for cytocompatibility tests was performed using the free Real Statistics Resource Pack software Release 7.2^59^. After checking the prerequisites, namely, a similar population variance, a normal distribution, independent samples and equal sample sizes, a one-way analysis of variance (ANOVA) was performed. If the null hypothesis was rejected (p < 0.05), there is a significant difference between the groups, and a post-hoc Ryan, Einot, Gabriel, Welsh Studentized Range Q (REGWQ) test was performed to test if the difference between the groups is statistically significant. Results are presented as the mean value ± the standard deviation (SD), in the Supporting Information (Figure S1).

The statistical analysis for cell migration speed was performed using R^60^ with the lme4 package^61^, to conduct a linear mixed effect analysis of the dependence of migration distance from laser power. A detailed overview of linear mixed effect modeling and a discussion of its benefits has been reported in literature^62^. We used time and laser power as fixed effects and the observed sample as a random effect. Visual inspection of the residuals did not reveal macroscopic deviations from normality and homoscedasticity. A likelihood ratio test of the full model against the same model without the effect of laser power was performed to obtain the p-value. Rejecting the null hypothesis (p < 0.05) indicates that laser power has a non-negligible influence on migration distance in time and therefore on migration speed.

## Results

### Multi-photon induced modifications in cell-laden hydrogels

**Figure 1:**
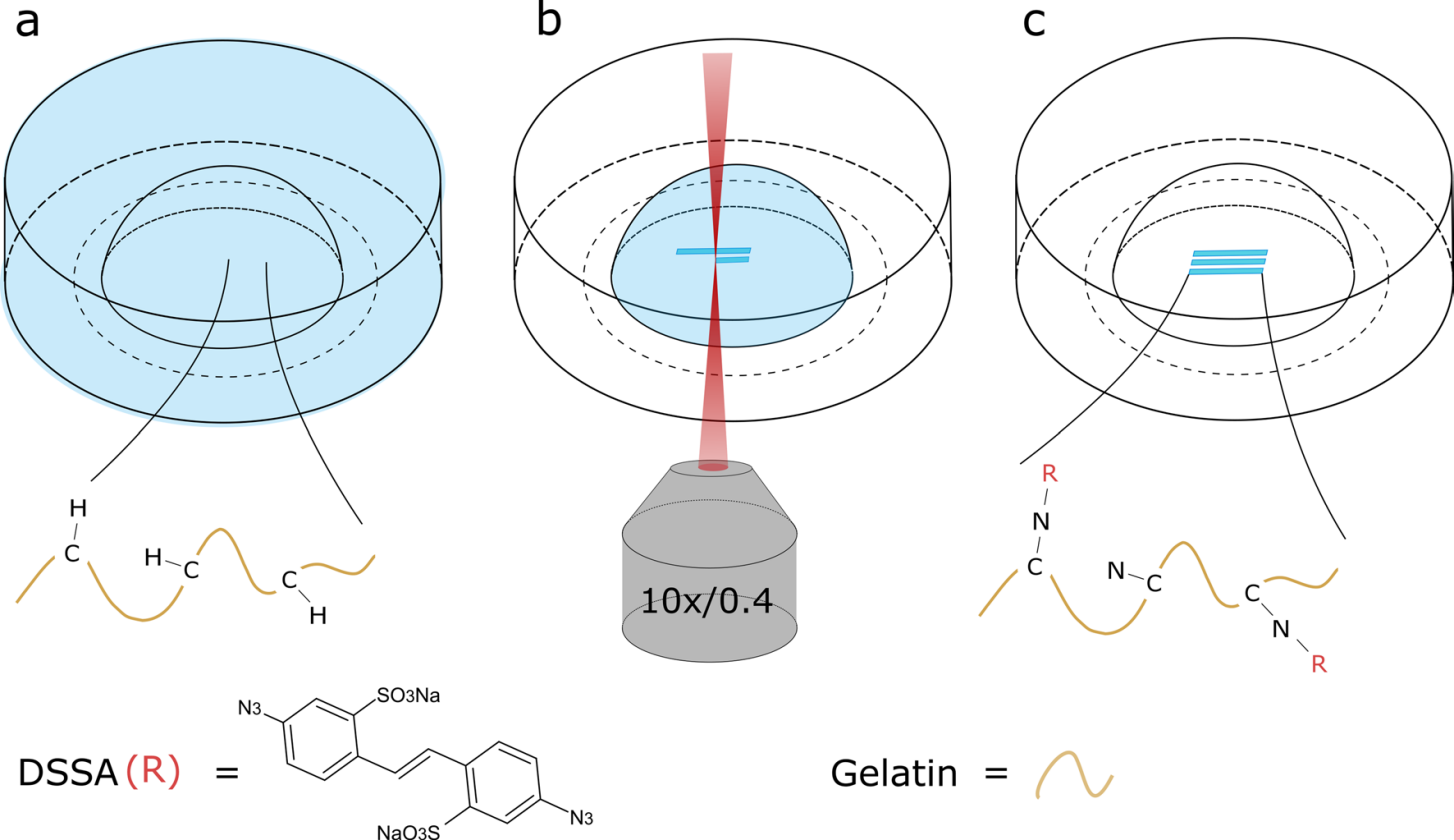
Schematic illustration of the DSSA multi-photon photografting process. (**a**) The UV-crosslinked gel-MA hydrogel pellet is soaked in a DSSA solution for 24 h. (**b**) Upon laser irradiation the azido group is group is photochemically decomposed into reactive nitrenes, (**c**) which covalently bind to C–H groups of gel-MA.

A schematic illustration of the MPL photografting process is depicted in Fig. 1. After incubating the hydrogel pellet for 24 h in a 1 mM DSSA solution, to ensure complete diffusion of the photografting agent (Fig. 1a), the sample was transferred to the MPL setup (Fig. 1b). Upon multi-photon absorption, the azido group is photochemically decomposed into reactive nitrenes, which immediately form covalent bonds with adjacent C–H functional groups of the gelatin backbone (Fig. 1c). This mechanism is universally applicable to a wide range of transparent and sufficiently dense hydrogels having C–H or N–H bonds^52^. Data from a PrestoBlue cell viability assay shows that the impact of the DSSA grafting agent on hASCs and HUVECs is not substantial (see Figure S1). Wavelengths of 700 and 725 nm have been used. Longer wavelengths have increased the grafting threshold only slightly. Since DSSA contains two azido groups, every molecule can potentially form two chemical bonds. Preliminary atomic force microscopy (AFM) results suggest that the hydrogel is becoming less stiff upon DSSA photografting. However, at expected values below 10 Pa, the hydrogel is too soft for reliable AFM measurements at an appropriate scale. We hypothesize that the photografted regions become more hydrophilic and therefore experience a decrease in stiffness. DSSA photografting has been identified as the relevant process behind the directional cell migration in a negative control experiment (Figure S2).

### Influence of photografted patterns on hASCs behavior

#### Alignment of hASCs

**Figure 2:**
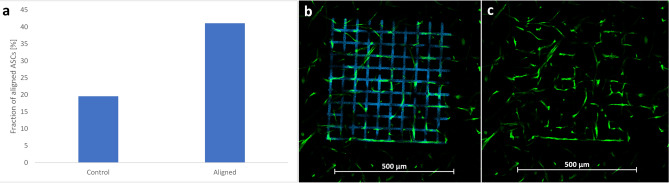
(**a**) Single image quantification of the orientation of hASCs in response to a photografted pattern on day 13. (**b** and **c**) GFP-hASCs encapsulated at a density of 1000 cells per 1 µL in a gelatin-based hydrogel. Image was taken on day 13 after photografting. In image (**b**) the grafted structure is fluorescing in blue. This signal is suppressed in image (**c**) to highlight the cellular alignment. The scale bars represent 500 µm.

To study the response of hASCs to DSSA-modified patterns, a mesh-like structure has been photografted within a cell-laden gel-MA pellet and the cell behavior has been observed over the course of 13 days. The structure consists of 10 parallel elongated cuboids (feature size of 10 µm × 10 µm, separated by 50 µm), superimposed by 10 parallel elongated cuboids, rotated at a 90-degree angle. Alternative mesh-like designs have shown that a decrease of either the feature size or the separation distance results in less cell alignment. Fluorescent images visualizing the alignment on day 13 after grafting are displayed in Fig. 2. The modified regions are fluorescing in blue and the hASCs are fluorescing in green. By directly comparing the images with and without the blue-fluorescent channel (Fig. 2b and c), the alignment of hASCs is highlighted. The preferential horizontal and vertical orientation of the hASCs is well visible in Fig. 2c. To facilitate the interpretation of the images, the orientation of hASCs has been quantified from the presented image. The results (Fig. 2a) are in agreement with the observed patterns, indicating that roughly 41% of cells are oriented horizontally and vertically, as compared to roughly 20% in the non-grafted control. This indicates that the cells next to the modified regions can sense the change in the material properties and respond accordingly by orienting their shape in alignment with the given pattern.

#### Directional migration of hASCs

Co-culture spheroids of hASCs and HUVECs in a 1 to 1 ratio have been encapsulated in gel-MA and the migration has been studied by means of laser scanning microscopy, in an effort to analyze the influence of the hydrogel modifications on the migration direction of cells.

**Figure 3:**
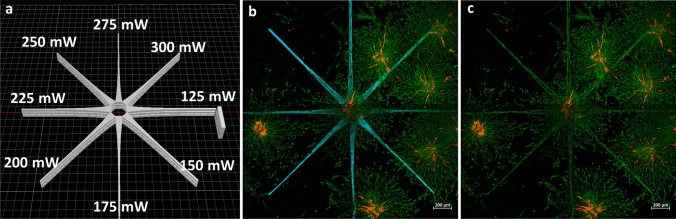
(**a**) Layout of the photografted structure and indication of the different power settings. (**b** and **c**) Laser scanning microscopy maximum intensity projection (MIP) images of co-culture spheroids of GFP-hASCs and RFP-HUVECs in a 1:1 ratio, encapsulated in a gelatin-based hydrogel. Images were taken on day 3 after photografting. The blue-fluorescent channel is not shown in image (**c**) to better highlight hASCs alignment along the grafted regions. The scale bars represent 500 µm. A time-lapse of GFP-hASC migration over the course of 61 h is shown in Video S1.

A design that ensures a high coverage of the spheroid’s vertical surface and thereby maximizes the ratio between guided and random migration has been created (Fig. 3a). It consists of 24 elongated isosceles trapezoidal-like structures (largest cross section: 100 µm × 30 µm; smallest cross section: 20 µm × 30 µm) arranged in three star-shaped layers. The isosceles trapezoidal-like patterns have been produced at different power settings for each direction (between 125 and 300 mW with a stepwise increase of 25 mW; Fig. 3a), to study the influence of printing power on directional cell migration. A cuboid has been grafted to denote the structure with the lowest power and to subsequently indicate the direction of increasing laser power.

In Fig. 3a, the grafted star-shaped pattern, fluorescing in blue, is wrapped around the co-culture spheroid located at the center of the image. The images have been taken on day 3 after grafting and depict a maximum intensity projection (MIP) of a z-stack taken along the vertical diameter of the spheroid. An image showcasing the 3D nature of the pattern, photografted around a spheroid, is displayed in Figure S3. Looking solely at the green-fluorescent signal in Fig. 3c, the grafted star-shaped structure becomes clearly visible, confirming that the hASCs have migrated into the pre-defined regions. Areas where the photografted structure is in contact with other spheroids on the same plane exhibit a higher cell density. This effect is especially pronounced in the top right region. A control experiment was performed to test the possibility that laser-induced material modifications beyond DSSA photografting are influencing the migration of hASCs. The results presented in Figure S2 show a preferential migration of cells in the DSSA grafted sample, compared to a random migration in the DSSA free control.

**Figure 4:**
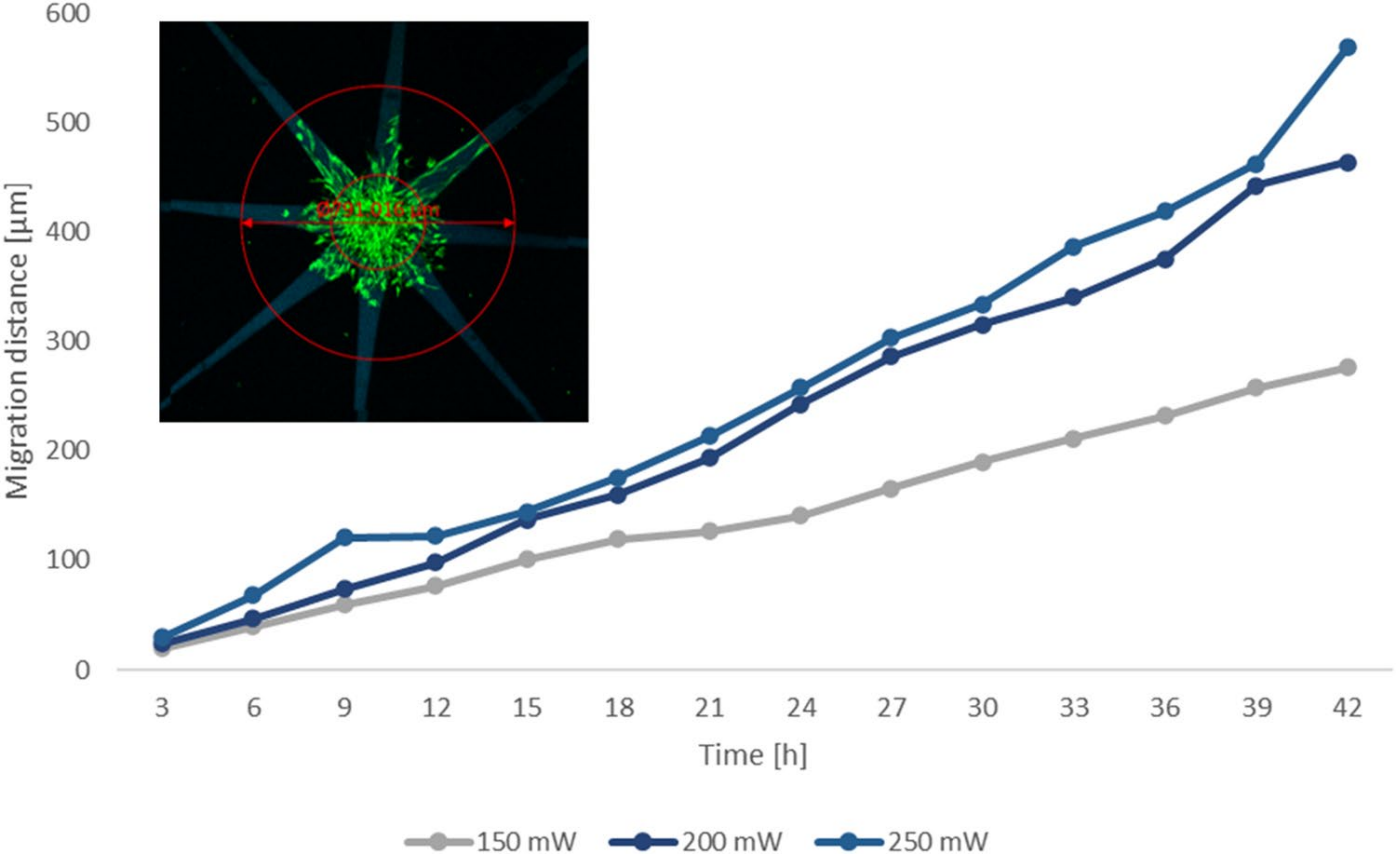
Quantification of hASCs migration: The migration distance of GFP-hASCs into the patterns, photografted at different power settings, is plotted over the time and calculated as a mean value from five samples in total and at least three different data sets per time point. A linear mixed effects model was used to interpret the data, due to the large deviation between samples. The image in the upper left quarter depicts how the migration distance was measured.

To study the potential correlation between laser power and cell migration, time-lapse imaging was performed over the course of 42 h. A video showcasing the migration of hASCs into the photografted patterns is displayed in Video S1. The recorded migration distance has been plotted over time for selected power values in Fig. 4. Since there was a large variability between the observed spheroids, we used a linear mixed effect model to quantify the influence of both time and laser power on cell migration distance, considering the random effects due to the variability between individual samples. The model indicated a strong effect of time on migration distance, coherent with the pictures we acquired and with the data in Fig. 4, and a weaker but still positive effect of power. The likelihood ratio test we performed against a model in which the parameter of power was excluded, confirmed that this effect is statistically relevant. This indicates that higher laser powers lead to an increase in the density of covalently bound DSSA molecules, which in turn induces a faster directional hASC migration. The cell behavior can therefore be controlled through the photografting parameters.

### Influence of photografted patterns on HUVEC behavior

Various iterations of the setup have been assessed for the potential to induce directional endothelial sprouting. In the scope of this article, only the optimized setup with regard to the material, cell and photografting parameters is presented.

This optimization process has led to interesting observations, the most prominent being that a sufficient number of hASCs have to be present to induce HUVEC sprouting. This appears to be the case for spheroids that are either in close proximity to other spheroids or are located in the bottom region of the hydrogel pellet, since the proliferation of hASCs on the glass surface seems to be substantially higher compared to the bulk. Therefore, the location of the spheroid within the hydrogel has a significant impact on the sprouting behavior. Since the current experimental design does not allow for the manual placement of spheroids, a high number of samples have been grafted and subsequentially observed under an LSM. Our results indicate that a 1 to 1 ratio is best suitable for hASCs guided migration as well as HUVEC sprouting. Although the total amount of HUVECs positively scales with an increasing HUVEC fraction, the number of observed sprouts is decreasing. This might be due to the dependence of endothelial sprout formation on the presence of hASCs. Similarly, a reduction in sprouts was also observed with increasing hASCs fractions, indicating that a certain amount of HUVECs have to be present for sprouting to occur. Various gel-MA concentrations between 5 wt% and 20 wt% have been tested. With increasing gel-MA concentration we could observe that the number of random as well as guided sprouts is declining. A certain hydrogel concentration is necessary to ensure structural integrity, therefore the 5 wt% concentration has been identified as best suitable for the present work.

**Figure 5:**
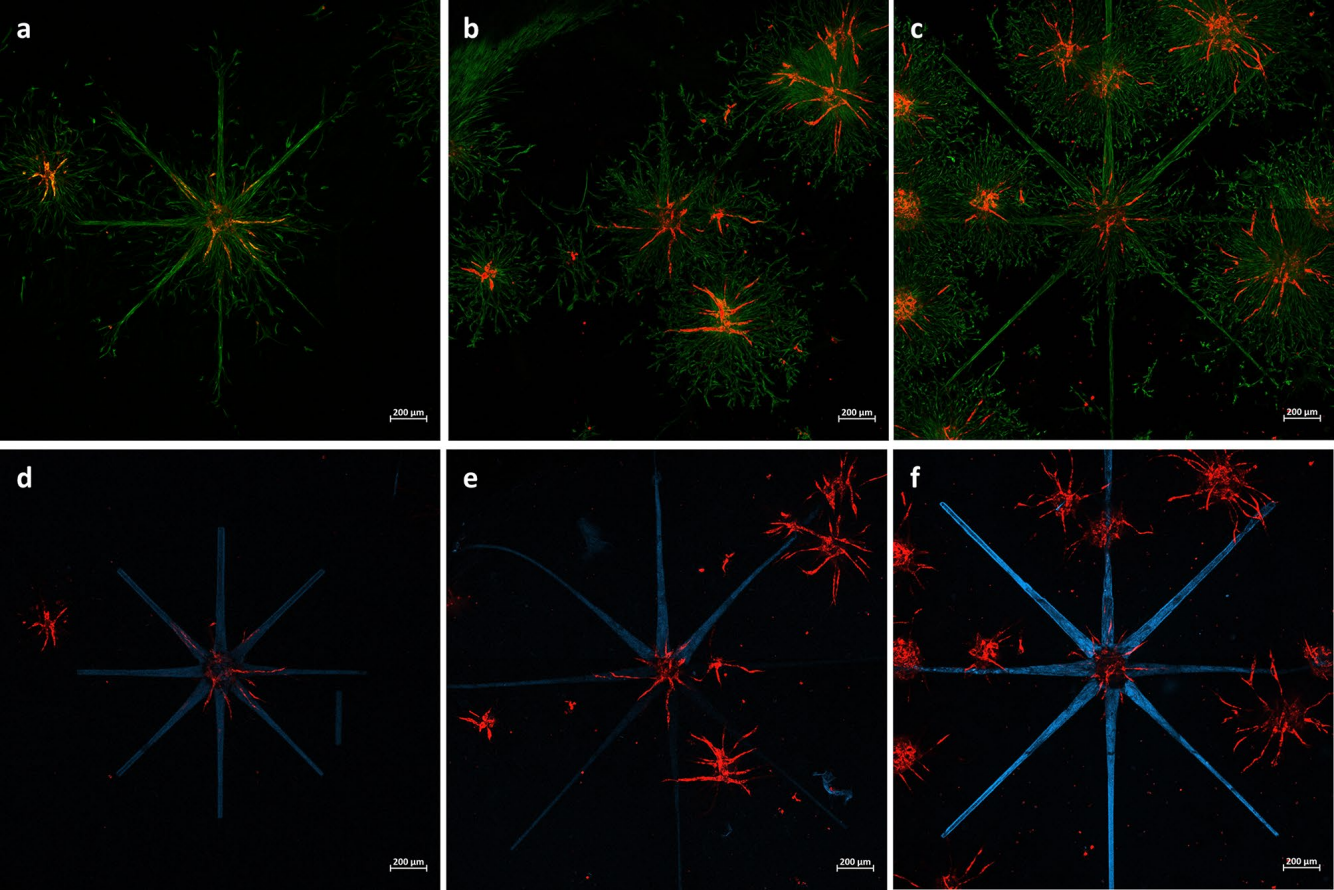
Laser scanning microscopy maximum intensity projection (MIP) images of RFP-HUVECs sprouting: Co-culture spheroids of GFP-hASCs and RFP-HUVECs in a 1 to 1 ratio, encapsulated in 5 wt% gel-MA DS 63. The photografted patterns are fluorescing in blue, the GFP-hASCs in green and the RFP-HUVECs in red. Every column displays the same sample, with the upper panel showing the green and red fluorescent channel to illustrate the dependence of sprouting on the positions of GFP-hASCs and in the lower panel the green and red fluorescent channels are displayed to illustrate the directional sprouting in accordance with the photografted structure. Images (**a,b,d** and **e**) have been taken on day 5, images (**c** and **f**) have been taken on day 3. The scale bars represent 500 µm.

Three samples showing a directional orientation of the endothelial sprouts, in dependence on the presence of hASCs, are displayed in Fig. 5. The images in the upper panel show the hASCs fluorescing in green and the HUVECs fluorescing in red, to highlight the dependence of HUVEC sprouting on the presence of hASCs. The lower panel of Fig. 5 displays the grafted structure fluorescing in blue and the HUVECs fluorescing in red. Although multiple sprouts are oriented towards the grafted regions, random sprouting is still occurring. Given the limited sample size of six and the presence of random sprouts, it is not possible to perform a statistical evaluation and hence to draw a definite conclusion.

The fluorescent signal intensity of the photografted patterns is lower in Fig. 5d and e compared to Fig. 5f. Since the intensity is higher for the sample imaged at an earlier time point after grafting, it is possible that the signal fades out over time. This could also be caused by a higher number of cells in the beam path, different positions of the spheroid within the hydrogel pellet or different settings of the confocal microscope. The pattern shown in Fig. 5d was adapted and elongated for subsequent samples (Fig. 5e and f), to increase the length of the migration path.

## Discussion

Many of the previously reported biofabrication methods for creating spatially organized microvessels in vitro exhibit significant shortcomings in terms of biocompatibility, resolution and the complexity of structures^38,40,41^. MPL enables structurally complex modifications at a high resolution inside 3D cell-laden hydrogels and presents a suitable alternative that overcomes these drawbacks. Previous applications of MPL as a means of altering the physico-chemical properties relied on materials with inherent specific functional groups^30,44–46^, with the exception of photoablation, for which the potential adverse effects of high laser powers remain to be addressed^48,49,63^.

We have presented a novel, widely applicable and biocompatible method that utilizes MPL to locally introduce DSSA molecules into the backbone of a polymer matrix. During the photografting agent selection process, we have identified certain criteria that need to be fulfilled. The molecule has to have azido groups so that it can be photografted, it has to be commercially available to ensure ease of use, it has to be cytocompatability and it has to be efficiently processable with MPL. DSSA-photografting can be performed with commercially available materials and is applicable to a wide range of hydrogels containing C–H or N–H bonds at any stage of a 3D tissue culture, without the necessity to adapt the matrix formation and cell encapsulation protocol. It therefore enables full spatial and temporal control over the modification process. With a laser scanning speed of 1000 mm/s, this versatile method allows for the creation of complex high-resolution 3D patterns at comparably high throughput. Additionally, multi-photon photografting could be combined with other biofabrication techniques and other MPL processes operating at a different wavelength. From a technical perspective, the feature size of photografted patterns can be reduced below 10 µm with the microscope objective that has been used in the present work. Moreover, by utilizing objectives with a higher magnification, the achievable resolution will increase accordingly, at the expense of the scanning speed. The cellular response, however, remains to be studied, and would probably not particularly benefit from a resolution increase in this case, because the features would be much smaller than the cell size.

Photografting could be applied to study processes that are influenced by spatial and temporal changes of the ECM stiffness. Suitable cell types can be encapsulated in corresponding hydrogels and the chemical composition can be locally altered through fs-laser irradiation. Relevant processes include the reprogramming of cells into tumor precursor cells^64^, metastasis^65,66^, fibrosis^67^ or wound healing^68^.

By altering the chemical properties of a cell-laden hydrogel through MPL grafting, we have demonstrated for the first time that hASCs preferentially align to photografted regions at the micrometer scale (Fig. 2). Quantitative analysis revealed that roughly 41% of cells are oriented in accordance with the modified regions on day 13 as opposed to 20% in the control group. These findings highlight the suitability of this novel method to adapt the microenvironment of cells.

Co-culture spheroids of hASCs and HUVECs have been encapsulated in gel-MA and a star-shaped pattern has been photografted around them. Consequently, the migration of cells has been observed with an LSM over the course of three days (Fig. 3). We were able to show that hASCs are preferentially migrating into the photografted patterns. The migration of cells is dependent on various characteristics of the surrounding matrix, such as the pore size, stiffness or biochemical cues^69,70^. Due to the hydrophilicity of DSSA, photografted patterns are expected to experience a higher degree of swelling compared to non-grafted regions. This most likely affects the pore size, the stiffness and the density of complete cell culture medium. A combination of those, among other factors, could be the driving force behind the directional hASC migration in the present model.

Time-lapse imaging allowed us to plot the migration distance over time for a set of increasing laser power values and a consequential trend of higher migration speeds was visualized (Fig. 4). Statistical analysis revealed that there is indeed a positive correlation between the laser power and the migration distance. This suggests that the laser power, which can be adapted arbitrarily within the printing procedure, is a suitable parameter to control the extent of the material modification.

Utilizing co-culture spheroids enabled us to study the influence of DSSA-modified patterns on endothelial sprouting. The present work outlines the potential of guiding vascularization via multi-photon photografting. It has previously been successfully demonstrated that co-culture spheroids of hASCs and HUVECs form a naturally organized network in vitro, capable of anastomosis with the vasculature of the host^71^.

Blood vessels consist of two cell types, endothelial cells (ECs) forming the inner layer and perivascular cells wrapped around them, that are interacting with each other. Besides providing structural support and regulating the permeability, perivascular cells are communicating with ECs through direct contact and paracrine signaling and are therefore influencing the proliferation, survival, migration and differentiation of ECs^72,73^, making them indispensable for the formation of stable vessels^74^. It has been reported that the presence of ASCs as a supporting cell type enhances vessel formation in vitro^75^. A study by Rohringer et al. (2014) has shown that ECs are capable of forming a network within a fibrin hydrogel upon the introduction of ASCs. This thorough investigation came to the conclusion that ASCs are secreting pro-angiogenic and regulatory proteins and that their presence is, therefore, necessary for the formation of endothelial sprouts^76^. Another study by Song et al. (2016) came to a similar conclusion and they identified the ASC-mediated matrix metalloproteinase (MMP)-dependent collagen remodeling as a crucial factor for inducing endothelial sprouting^77^. A large number of publications are focusing on the interplay of ASCs and HUVECs. A good overview is given in the review paper by Rautiainen et al. (2021), which highlights the importance of direct contact between ECs and ASCs for endothelial network formation^78^. Based on these publications, it seems like hASCs are supporting HUVEC migration by providing chemical cues and by creating degraded pathways within the hydrogel.

In accordance with these results, we have observed that HUVEC mono-cultures encapsulated in gel-MA are not capable of migrating into the hydrogel. Due to the strong dependence of HUVEC migration on the presence of hASCs, we hypothesize that the sprouting direction of HUVECs is dependent on the spatial distribution of hASCs.

To maximize the ratio between guided and random hASC migration, a grafting design with 24 elongated isosceles trapezoidal-like structures (cross-section: largest 100 µm × 30 µm, smallest 20 µm × 30 µm), arranged in three star-shaped layers, to ensure full coverage of the spheroid’s vertical surface, displayed in Fig. 3a, has been created.

We have observed a strong inhomogeneity in the overall number and length of endothelial sprouts between different samples, which can be partially explained by the dependence of HUVECs on hASCs. It seems that a sufficient amount of hASCs is necessary in order to induce HUVEC sprouting. This is the case if the spheroid of interest is in proximity to other spheroids or close to the bottom of the hydrogel pellet, where hASC proliferation and migration are high on the glass surface. It was observed that HUVECs are sprouting in the presence of a sufficient number of hASCs (Fig. 5). The spheroids displayed in Fig. 5 are all located in the bottom region of the hydrogel pellet. In the current setup, the spheroids are distributed randomly within the hydrogel. Therefore, a large number of samples would need to be photografted, analyzed and classified according to the locations of the spheroids, to identify statistical differences that are expected to be rather weak in the present case. Therefore, a reproducible method of spheroid placement remains to be established, before a statistical interpretation is possible. While it was possible to guide the hASCs into the grafted channels, which is indicated in Fig. 5 by the higher green-fluorescent intensity in areas corresponding to the photografted structure, there is still a pronounced random migration of hASCs. The upper panel images of Fig. 5 show that HUVEC sprouts are only located in areas where hASCs are present. This observation provides further supporting evidence on the dependence of endothelial sprouting on pro-angiogenic cytokines released by hASCs and is in accordance with various studies that have highlighted the importance of close contact between ECs and ASCs in endothelial network formation^78^. Although it seems possible to encourage HUVECs to sprout in pre-defined directions, random sprouting is inevitable due to the aforementioned random migration of hASCs. Given the indication that in the presented model HUVEC sprouting is dependent on the presence of hASCs, it should be possible to guide the migration of endothelial cells, once hASCs can be better confined to the grafted patterns.

To ensure reproducibility, the spheroids would need to be placed manually within the hydrogel, where they can then be connected via photografted patterns. Further efforts could be dedicated to establishing such a process that allows for the controlled placement of the spheroids. The culture conditions could be also more finely controlled by moving into a microfluidic chip or a bioreactor, where nutrients and growth factors concentrations, gradients and flow-induced shear stress could reduce the dependence on the signaling provided by the hASCs^79^. By combining the concept of co-culture spheroids as vascular building blocks with MPL, it could be possible to establish a vascular network through the precise positioning of cell spheroids^80^ and subsequent application of photografting to guide the formation of the microvasculature.

## Conclusion

We have presented a straightforward biofabrication technique that satisfies the requirements of biocompatibility, high-resolution and high complexity that are necessary for the creation of microvessels and the adaption of the microenvironment of individual cells. Furthermore, it is universally applicable and comparably fast, with a laser scanning speed of 1000 mm/s, and able to produce modifications inside the volume of an already crosslinked and cell-laden hydrogel. This method is based on multi-photon photografting and works by covalently binding DSSA molecules to C–H or N–H bonds from the polymer backbone of cell-laden hydrogel matrices. Special modifiable functional groups are not required and this technique is therefore applicable to any sufficiently dense, transparent hydrogel. DSSA-photografting can be performed at various stages of the cell culture process, without the need to fundamentally adapt existing protocols. The present work has shown that encapsulated hASCs are aligning to a mesh-like structure (feature size of 10 µm × 10 µm) upon photografting in a gelatin-based hydrogel. The method can therefore be used to study processes that are influenced by physico-chemical changes of the ECM, such as cell differentiation, proliferation and motility. By transferring this approach to an hASC and HUVEC spheroid co-culture system, we have demonstrated that HUVEC migration is dependent on the presence of hASCs and that it could therefore be possible to control the direction of endothelial sprouting by confining the hASCs to the grafted pattern. A more controllable process needs to be established to account for the heterogeneity of results between different samples. This work paves the way towards the use of this technique inside microfluidic devices, where the effect of further cellular signals, such as fluid flow-induced shear stress or vascular endothelial growth factor (VEGF) concentration gradients, can be studied and ultimately used for the controlled vascularization of organs-on-a-chip.

## Supplementary Information

Supplementary Information.

Supplementary Legends.

Supplementary Video S1.

## Data Availability

The datasets generated during and/or analyzed during the current study are available from the corresponding author on reasonable request.
