# Publisher Correction: Guiding cell migration in 3D with high-resolution photografting

**DOI:** 10.1038/s41598-022-14892-6

**Published:** 2022-06-17

**Authors:** Simon Sayer, Tommaso Zandrini, Marica Markovic, Jasper Van Hoorick, Sandra Van Vlierberghe, Stefan Baudis, Wolfgang Holnthoner, Aleksandr Ovsianikov

**Affiliations:** 1 Research Group 3D Printing and Biofabrication, Institute of Materials Science and Technology, TU Wien, Vienna, Austria; 2 Austrian Cluster for Tissue Regeneration (https://www.tissue-regeneration.at), Vienna, Austria; 3 Polymer Chemistry and Biomaterials Group, Centre of Macromolecular Chemistry, Department of Organic and Macromolecular Chemistry, Ghent University, Ghent, Belgium; 4 Polymer Chemistry and Technology Group, Institute of Applied Synthetic Chemistry, TU Wien, Vienna, Austria; 5 Ludwig-Boltzmann-Institute for Traumatology, The Research Centre in Cooperation with AUVA, Vienna, Austria

Correction to: *Scientific Reports* https://doi.org/10.1038/s41598-022-11612-y, published online 23 May 2022

The original version of this Article contained a typographical error.

Figure 1(b) did not display correctly.

**Figure 1:**
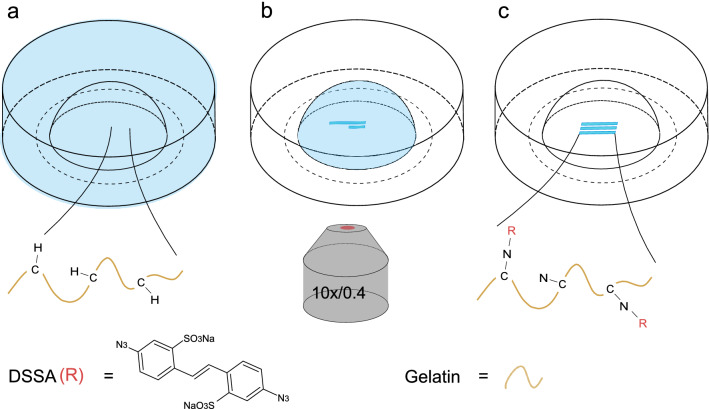
Schematic illustration of the DSSA multi-photon photografting process. (**a**) The UV-crosslinked gel-MA hydrogel pellet is soaked in a DSSA solution for 24 h. (**b**) Upon laser irradiation the azido group is group is photochemically decomposed into reactive nitrenes, (**c**) which covalently bind to C–H groups of gel-MA.

The original Figure 1 and accompanying legend appears below.

The original Article has been corrected.

