# Supplementary material for: Guiding cell migration in 3D with high-resolution photografting
Source: Sci Rep. 2022 May 23;12:8626. doi: 10.1038/s41598-022-11612-y (PMC9126875; doi:10.1038/s41598-022-11612-y)
Supplement: Supplementary file 1 — Supplementary Information. [file 41598_2022_11612_MOESM1_ESM.pdf]

Supporting Information
Guiding cell migration in 3D with high-resolution photografting

Simon Sayer[1,2], Tommaso Zandrini[1,2], Marica Markovic[1,2], Jasper Van Hoorick[3], Sandra Van Vlierberghe[3], Stefan Baudis[4,2], Wolfgang Holnthoner[5,2], Aleksandr Ovsianikov[1,2*]

[1]3D Printing and Biofabrication Group, Institute of Materials Science and Technology, Research Unit of Polymers and Composites, TU Wien, Vienna, Austria

[2]Austrian Cluster for Tissue Regeneration

[3]Polymer Chemistry and Biomaterials Group, Centre of Macromolecular Chemistry, Department of Organic and Macromolecular Chemistry, Ghent University, Ghent, Belgium

[4]Polymer Chemistry and Technology Group, Research Unit Macromolecular Chemistry, Institute of Applied Synthetic Chemistry, TU Wien, Vienna, Austria

[5]AUVA Research Centre, Ludwig Boltzmann Institute for Experimental and Clinical Traumatology Vienna, Austria

*** Correspondence:**
Aleksandr Ovsianikov
Aleksandr.Ovsianikov@tuwien.ac.at

Cytocompatibility of DSSA grafting agent

To test the cytocompatibility of the 4,4′-Diazido-2,2′-stilbenedisulfonic acid (DSSA, Sigma-Aldrich) grafting agent, a PrestoBlue cell viability reagent *(*Thermo Fisher Scientific*)* was used. DSSA was dissolved at a concentration of 4 mM in EGM-2 supplemented with 5% FCS and EGM-2 supplemented with 10% FCS for HUVECs and hASCs, respectively and then diluted 1:1 to achieve concentrations of 2 mM and 1 mM. The cells were seeded in a 96-well plate at a density of 5 000 cells per well and incubated for 24 hours to let them attach to the surface. Afterward, the medium was exchanged for different concentrations of DSSA (4, 2 and 1 mM). One plate per cell type was placed in a UV crosslinker (Boekel) and irradiated with 1 J at a wavelength of 365 nm. The others were put in the incubator. After 24 hours of incubation with the DSSA grafting agent, the PrestoBlue cell viability assay was performed. PrestoBlue was diluted at 1:10 in the adequate medium and 100 µL per well was added. After 1 hour of incubation, the fluorescent signal was measured with a plate reader (Synergy BioTek, excitation 560 nm, emission 590 nm). The values were corrected for the background fluorescent signal. Medium without diluted DSSA was used as a positive control and cells treated with 50% dimethyl sulfoxide (DMSO, Sigma-Aldrich) for 1 hour acted as the negative control. The effect of DSSA was compared to the positive control, which we assumed to have 100% metabolic activity.

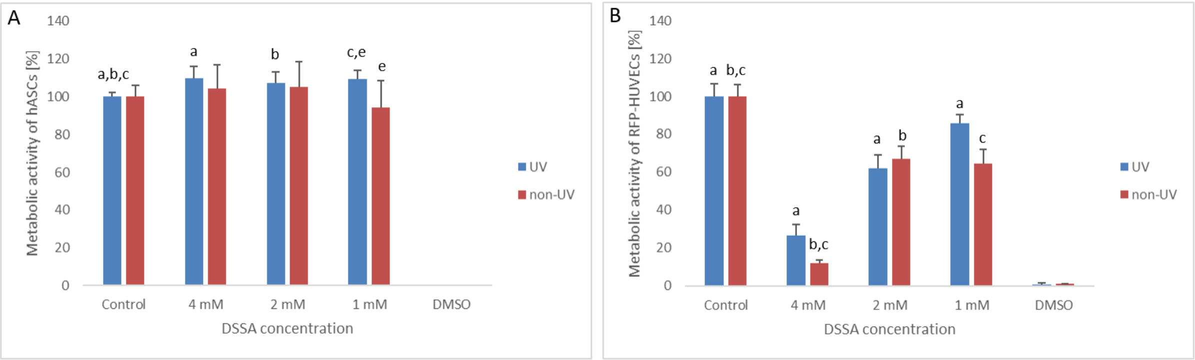

Figure S1: Determination of cytotoxic behavior of DSSA on (A) ASCs and (B) RFP-HUVECs using the PrestoBlue cell viability assay. Letters indicate a significant difference at $p < 0.05$ level. In graph (B), all values are significantly different in comparison to the control within the UV group and within the non-UV group, except for 2 mM and 1 mM.

The effect of the DSSA grafting agent on the metabolic activity of hASCs and HUVECs was tested for concentrations of 4, 2 and 1 mM with a PrestoBlue cell viability assay, which acts as an indication for the cytotoxicity of the grafting agent. As depicted in Figure S1A, hASCs exposed to DSSA and UV irradiation, show a significantly higher metabolic activity compared to the control. There is no significant difference between different DSSA concentrations. Exposure of hASCs to DSSA without UV irradiation shows no significant effect on the metabolic activity. For HUVECs, displayed in Figure S1B, there is a significant difference in comparison to the control for all tested concentrations, except for 2 and 1 mM in the non-UV irradiation group. The results indicate that the influence of DSSA and UV irradiation on hASCs is minor, whereas HUVECs are strongly affected. A concentration-dependent effect is visible for HUVECs, whereby the decrease in metabolic activity increases with higher DSSA concentrations. Therefore, with a metabolic activity of around 86%, the 1 mM concentration has been selected for the following experiments.

DSSA-free control experiment

A negative control experiment, in which a DSSA-free control sample has been subjected to multi-photon lithography, has been performed to test for potential material modifications, which may not be caused by DSSA photografting. The samples have been prepared as stated in the section Materials and Methods. One sample has not been soaked in the DSSA solution. A comparison of the cell migration in the photografted and the DSSA-free control samples is given in Figure S2. It shows that ASCs are preferentially migrating into the photografted pattern, while the migration in the DSSA-free control is random.

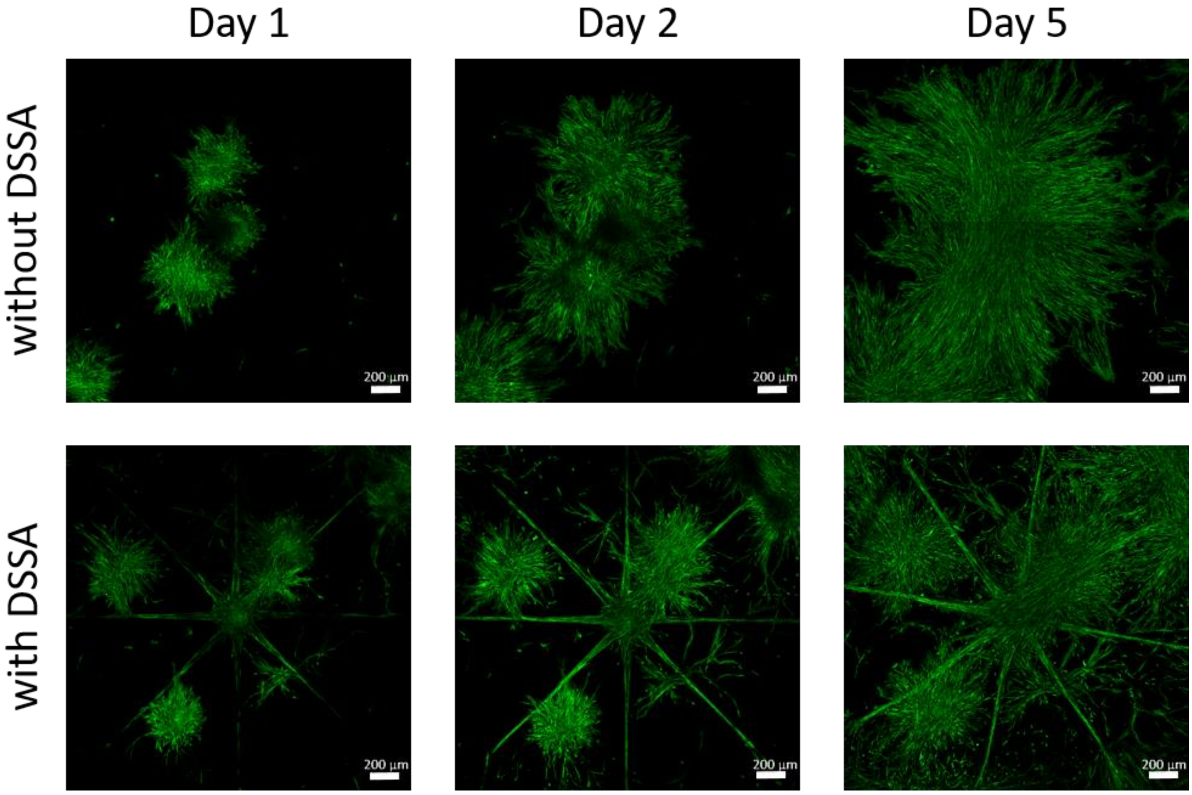

Figure S2: Maximum intensity projection (MIP) of GFP-hASC spheroids encapsulated in 5 wt% gel-MA DS 63 at days 1, 2 and 5 after laser patterning. The lower panel shows photografted samples, in which hASCs are following the grafted patterns from the central spheroid. The scale bars represent 200 µm.

3D image

3D images of a GFP-hASC spheroid encapsulated in 5% gel-MA taken on day five after photografting are shown in Figure S3. In Figure S3A, only the blue fluorescent channel, relating to the presence of grafted molecules, is shown, to further highlight the 3D nature of the grafted pattern. The pattern was grafted at the center of the spheroid to increase the fraction of guided hASCs. Separation of the rods in z-direction is only slightly visible, due to the limited z-resolution of the confocal microscope and interference of the subjacent rods.

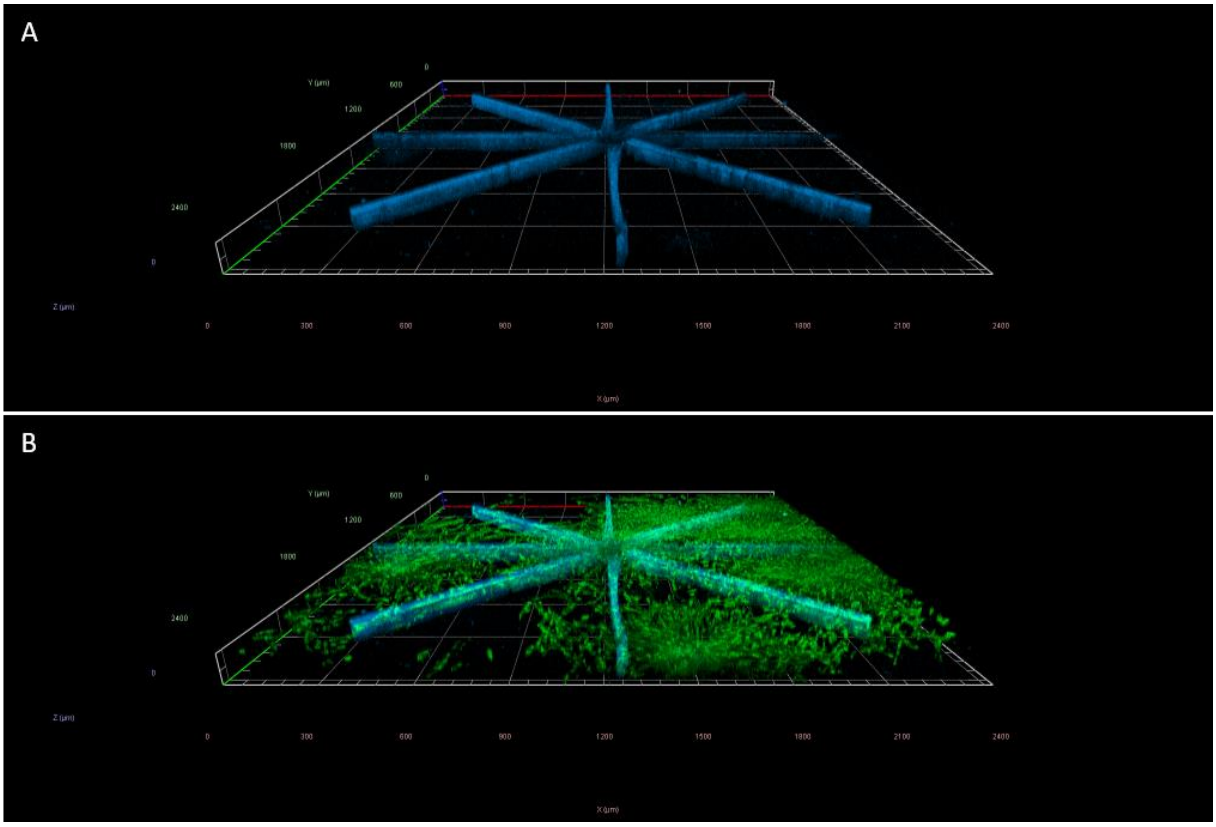

Figure S3: 3D image of GFP-hASC spheroids encapsulated in 5 wt% gel-MA DS 63 taken at day 5 after photografting. Image (A) shows solely the 3D grafted pattern fluorescing in blue. In image (B) the green fluorescent channel is added, showcasing the distribution of GFP-hASCs along the grafted pattern.

Time-lapse imaging and quantification of migration distance

The time-lapse imaging was performed at 37 °C and 5% CO_2, in a humid environment. The migration distances were quantified using the ZEN Blue 2.6 software. Distances were measured from the inner edge of the grafted pattern to the outermost fluorescent cell within the grafted pattern, at the respective time points. In Video S1 it is possible to observe the sequence of 27 images acquired over the period of 61 hours with time-lapse imaging. The migration of hASCs into the photografted patterns over time is well visible. In Figure S4, a photografted sample, taken 13.5 hours after encapsulation in a time-lapse imaging series, is displayed. The red measurement circle showcases how the migration distance was evaluated. The cell which has migrated the farthest, at a specific time point within the respective grafted sample, which was in this case the rod facing in the direction of the right corner and grafted with a laser power of 300 mW, was used as the reference to measure the distance. The inner diameter of the pattern, which is roughly equivalent to the spheroid size was subtracted from half of the measured value, to obtain the actual migration distance.

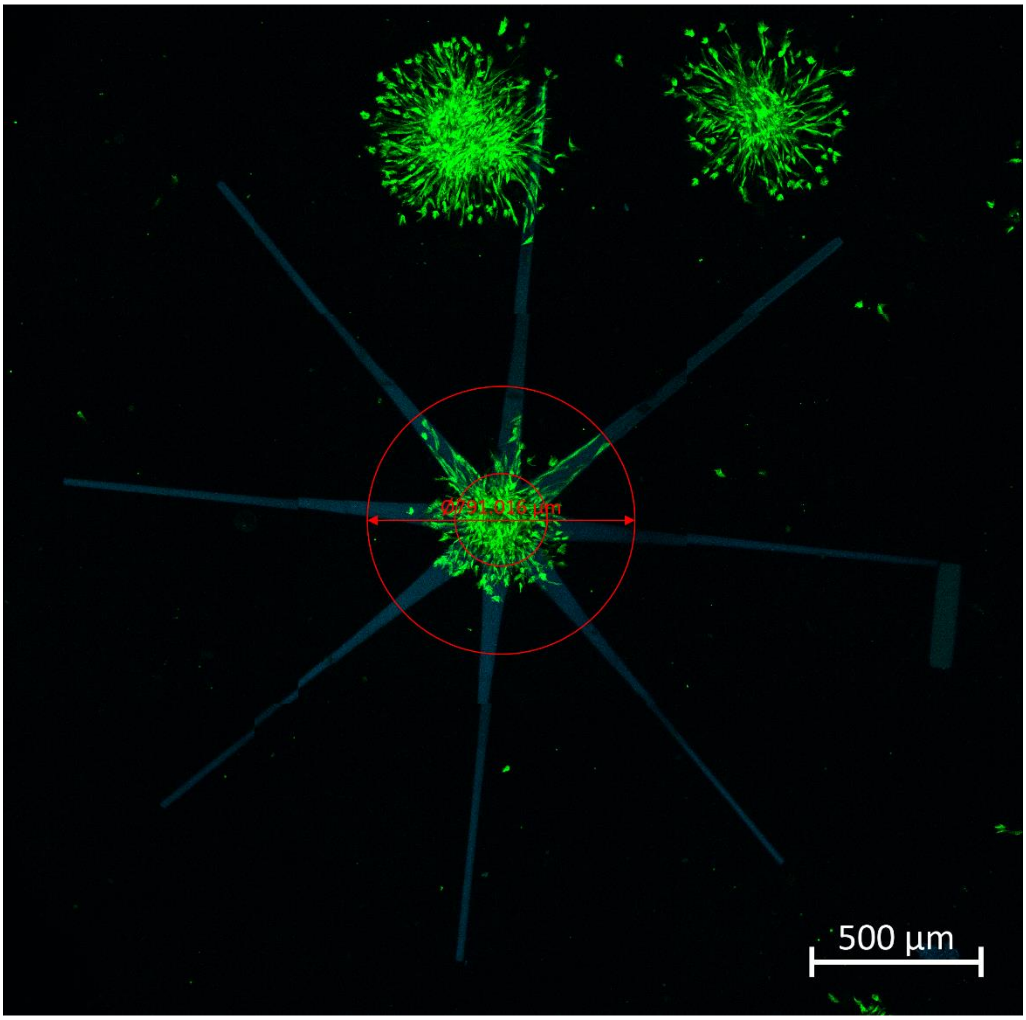

Figure S4: Maximum intensity projection (MIP) of GFP-hASC spheroids encapsulated in 5 wt% gel-MA DS 63, taken from a time-lapse imaging series at 13.5 hours after encapsulation. The photografted pattern is fluorescing in blue. The measurement circle showcases how the migration distance was evaluated. The scale bars represent 500 µm.

It can be seen in Figure S4 that the hASCs are preferentially migrating into the grafted pattern. In longer term experiments, which are necessary to observe HUVEC sprouting, the amount of migrating hASCs seems to be too high to ensure confinement to the pattern, resulting in cell migration into the surrounding hydrogel. Looking at the upwards facing rod in Figure S4, cells from a neighboring spheroid can be seen to migrate into the grafted pattern from the opposing side. In this case, data points were excluded as soon as the outwards and inwards migrating cells crossed their paths. This led to the reduction of data points for individual time points.

The migration distances at certain time points vary between the individual samples. This can be due to various reasons, such as the location of the spheroid within the hydrogel, the presence of neighboring spheroids or the variability between individual spheroids. It is important to note that the relative migration speed of cells in rods grafted at different laser powers is independent on the total migration speed which is largely dependent on the aforementioned factors. The standard deviation of the data is high and a statistical analysis is not straight forward. A linear mixed effect model was used to check the dependence of migration distance on the laser power. We used time and laser power as fixed effects and the observed sample as a random effect. Visual inspection of the residuals did not reveal macroscopic deviations from normality and homoscedasticity. A likelihood ratio test of the full model against the same model without the effect of laser power was performed to obtain the

p-value. Rejecting the null hypothesis ($p < 0.05$) indicates that laser power has a non-negligible positive influence on migration distance in time and therefore on migration speed.
