# Supplementary material for: Guiding cell migration in 3D with high-resolution photografting
Source: Sci Rep. 2022 May 23;12:8626. doi: 10.1038/s41598-022-11612-y (PMC9126875; doi:10.1038/s41598-022-11612-y)
Supplement: Supplementary file 2 — Supplementary Legends. [file 41598_2022_11612_MOESM2_ESM.docx]

Title: Time-lapse images of hASCs migrating into the DSSA photografted pattern.

*Video S1: Sequence of 27 images acquired over the period of 61 hours with time-lapse imaging. The migration of GFP-hASCs into blue fluorescent photografted pattern is displayed.*
